# The all age asthma cohort (ALLIANCE) - from early beginnings to chronic disease: a longitudinal cohort study

**DOI:** 10.1186/s12890-018-0705-6

**Published:** 2018-08-20

**Authors:** Oliver Fuchs, Thomas Bahmer, Markus Weckmann, Anna-Maria Dittrich, Bianca Schaub, Barbara Rösler, Christine Happle, Folke Brinkmann, Isabell Ricklefs, Inke R. König, Henrik Watz, Klaus F. Rabe, Matthias V. Kopp, Gesine Hansen, Erika von Mutius, the ALLIANCE Study Group as part of the German Centre for Lung Research (DZL)

**Affiliations:** 1 Dr von Hauner Children’s Hospital, Ludwig Maximilians University, Munich, Germany; 2 Department of Paediatric Respiratory Medicine, Inselspital, University Children’s Hospital of Bern, University of Bern, Bern, Switzerland; 3 Division of Pediatric Pulmonology and Allergology, University Children’s Hospital, Luebeck, Germany; 4 LungenClinic Grosshansdorf GmbH, Grosshansdorf, Germany; 5 Department of Paediatric Pneumology, Allergology and Neonatology, Hannover Medical School, Hannover, Germany; 6 Department of Paediatric Pneumology, University Children’s Hospital, Ruhr-University Bochum, Bochum, Germany; 7 Institute for Medical Biometry and Statistics, University Luebeck, University Medical Centre Schleswig-Holstein, Campus Luebeck, Luebeck, Germany; 8 Pulmonary Research Institute at LungenClinic Grosshansdorf , Grosshansdorf, Germany; 9 Institut für Asthma- und Allergieprävention (IAP), Helmholtz Zentrum Munich, Deutsches Forschungszentrum für Gesundheit und Umwelt (GmbH), Munich, Germany; 10 Comprehensive Pneumology Center - Munich (CPC-M), German Center for Lung Research (DZL), Munich, Germany; 11 Airway Research Center North (ARCN), German Center for Lung Research (DZL), Grosshansdorf, Germany; 12 Biomedical Research in Endstage and Obstructive Lung Disease Hannover (BREATH), German Center for Lung Research (DZL), Hannover, Germany

**Keywords:** Wheeze, Asthma, Pediatric pulmonology, Phenotype, Endotype, Biomarker

## Abstract

**Background:**

Asthma and wheezing disorders in childhood and adulthood are clinically heterogeneous regarding disease presentation, natural course, and response to treatment. Deciphering common disease mechanisms in distinct subgroups requires harmonized molecular (endo-) phenotyping of both children and adult patients with asthma in a prospective, longitudinal setting.

**Methods:**

The ALL Age Asthma Cohort (ALLIANCE) of the German Center for Lung Research (DZL) is a prospective, multi-center, observational cohort study with seven recruiting sites across Germany. Data are derived from four sources: (a) patient history from medical records, (b) standardized questionnaires and structured interviews, (c) telephone interviews, and (d) objective measurements. Objective measurements include amongst others lung function and quantitative assessment of airway inflammation and exhaled breath, peripheral blood, skin, nasal, pharyngeal, and nasopharyngeal swabs, nasal secretions, primary nasal epithelial cells, and induced sputum. In cases, objective measurements and biomaterial collection are performed regularly, while control subjects are only examined once at baseline.

**Discussion:**

The standardized and detailed collection of epidemiological and physiological data, and the molecular deep phenotyping of a comprehensive range of biomaterials in a considerable number of study participants across all ages are the outstanding characteristics of this multi-center cohort. Despite extensive biomaterial sampling, and a recruitment strategy that also includes pre-school children as young as 6 months, attrition is low. In children 83.9%, and in adults 90.5% attended the 12-month follow-up. The earliest time-point to include cases, however, is disease manifestation. Therefore, unraveling mechanisms that drive disease onset is limited, as this question can only be answered in a population-based birth cohort. Nonetheless, ALLIANCE offers a unique, integrative and inter-disciplinary framework with a comprehensive molecular approach in a prospective and identical fashion across ages in order to identify biomarkers and predictors for distinct childhood wheeze and asthma trajectories as well as their further course during adulthood. Ultimately, this approach aims to translate its most significant findings into clinical practice, and to improve asthma transition from adolescence to adulthood.

**Trial registration:**

NCT02496468 for pediatric arm, NCT02419274 for adult arm.

## Background

Wheezing disorders and asthma are the most prevalent chronic respiratory diseases both in childhood and adulthood. About 25–30% of children have at least one episode of wheeze before their 3rd birthday, but considerable clinical heterogeneity exists [1, 2]. Many of these children become symptom-free between 3 and 8 years of age, but some go on to persistent asthma in later childhood and adulthood [3].

**Fig. 1:**
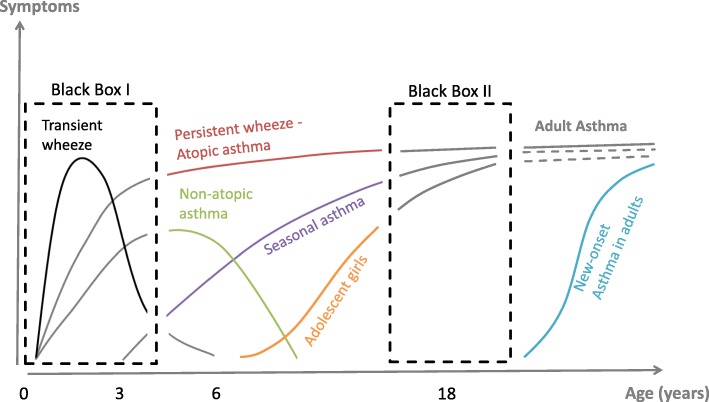
Wheeze and asthma phenotypes during childhood and adulthood. About 25–30% of children have at least one episode of wheeze before their 3rd birthday, but considerable clinical heterogeneity exists (broken line box I). Children with transient wheeze become symptom-free before school-age, those with non-atopic asthma after about 8 years of age. However, some, especially those with persistent atopic wheeze and seasonal triggers of wheeze go on to persistent asthma in later childhood and adulthood. Interestingly, girls present with new-onset asthma in significant numbers during adolescence, thereby adding to turning the sex-based bias from male towards female sex. While there is also new-onset-asthma during adulthood, it is unclear whether differences between persistent childhood asthma phenotypes continue throughout transition (broken line box II) into adulthood

Although the characteristic clinical manifestations of asthma in children and in adults are rather uniform with wheezing, shortness of breath and cough, population-based clinical and genetic studies suggest that asthma is not one disease but many [4]. Despite its prevalence, little is known about the diverse underlying pathomechanisms determining the different asthma phenotypes both in children and adults, including asthma transition (Fig. 1) [3–6]. Affected individuals vary with regard to severity and nature of their primary complaints, but also in relation to comorbidities, response to treatment, and to the course of the disease throughout life [2]. The different asthma phenotypes and trajectories are presumably caused by diverse underlying pathophysiological processes. Recent related research also takes complex, explicit molecular data into account to better link biology to clinical presentation which may then be called an asthma endotype [5, 7].

So far, most (endo)-phenotyping has been applied in children and adults separately. Consequently, there is a substantial intrinsic bias towards more pathophysiological data from cross-sectional studies in adults and more epidemiological data for children, respectively [3]. However, common predictors and subsequent targeted treatment - or even prevention strategies - are urgently needed, especially early in life. Asthma research to date should therefore integrate standardized molecular approaches in identical ways in longitudinal studies in paediatric and adult populations [3, 8, 9]. The decoding of mechanisms underlying the asthma syndrome, and their translation to the individual patient across all ages is the overall aim of the All Age Asthma Cohort (ALLIANCE) of the German Centre for Lung Research (Deutsches Zentrum für Lungenforschung, DZL).

## Methods/design

### Where is the study located and how is it funded?

The ALLIANCE infrastructure is provided by the participating sites of the German Centre for Lung Research (DZL) and associated study centres, i.e. university hospitals, academic and private research institutions in Luebeck, Grosshansdorf, Borstel, Hannover, Munich, Marburg and Cologne. Direct costs of the ALLIANCE Cohort are being paid by project grants (first funding period 2011–2015 and second funding period 2016–2020) from the German Federal Ministry of Education and Research (Bundesministerium für Bildung und Forschung, BMBF) as part of the DZL funding. All studies were approved by the local ethics committees and are registered at *clinicaltrials.gov* (paediatric arm: NCT02496468; adult arm: NCT02419274).

### Who is in the cohort?

**Fig. 2:**
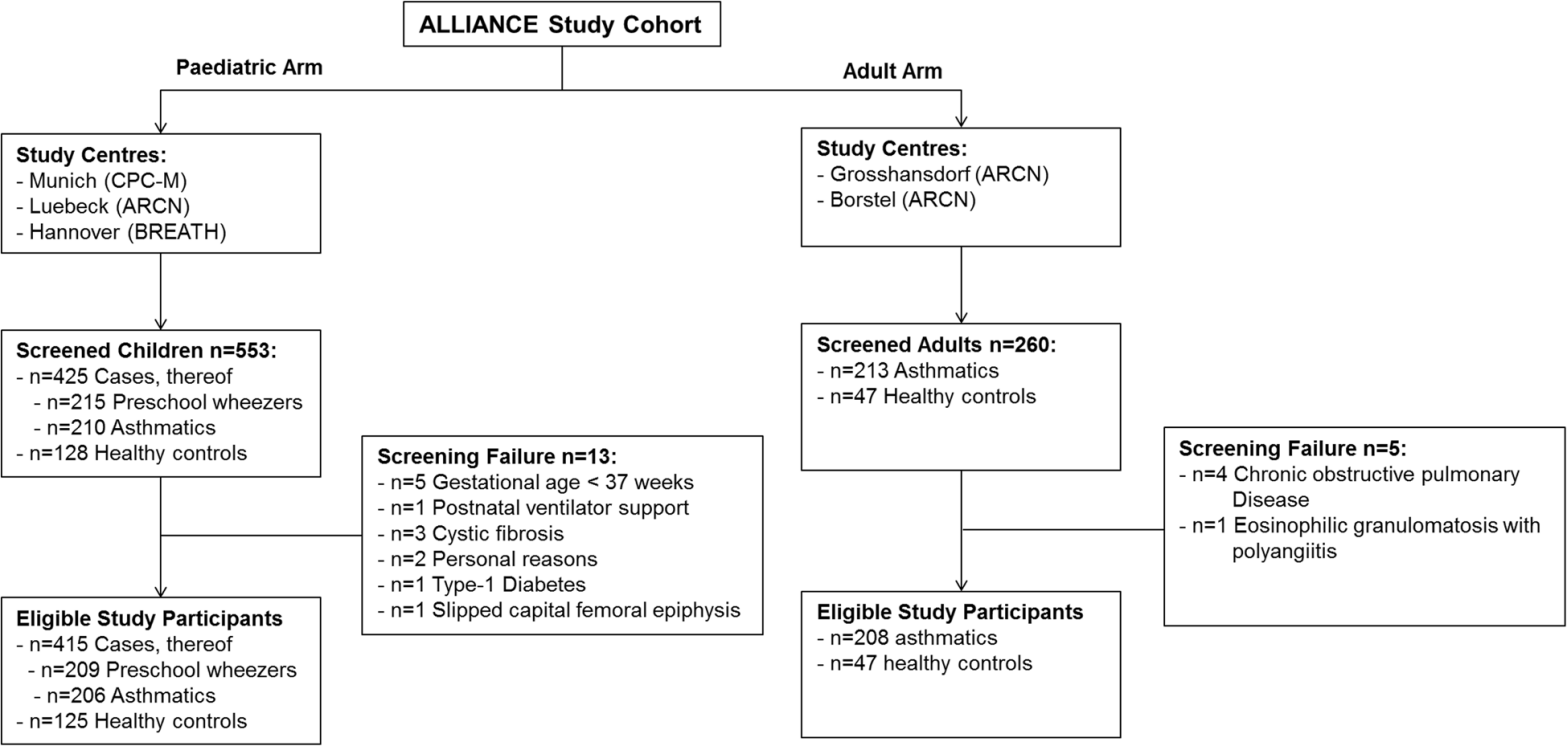
Consolidated Standards of Reporting Trials (Consort) Diagram displaying details for recruitment of participants of the DZL All Age Asthma Cohort (ALLIANCE). See text for details.

Study participants for both arms (paediatric and adult) of ALLIANCE are recruited at the participating study sites (for details see Fig. 2). In the case of children with new-onset disease, cases were additionally recruited in private practices of registered paediatricians. For these children, disease course can be followed from the beginning on, and they are rarely seen in a clinical but much more often in a primary care setting due to their early disease state. Healthy paediatric controls (age- and sex-matched) were recruited in the same centres, via notices across campus and in private practices of registered paediatricians, as well as during clinics of separate subspecialties (e.g. paediatric growth) or if scheduled for short surgical interventions.

Adult patients with asthma are recruited from the inpatient and outpatient departments of LungenClinic Grosshansdorf and of the Medical Clinical of Research Center Borstel, and from an institutional database that is used for clinical trials (Pulmonary Research Institute at LungenClinic Grosshansdorf). Healthy adult controls were also recruited from an existing institutional database and by advertisement.

### General in−/exclusion criteria for children, definition of childhood cases and controls

For children, the following inclusion criteria apply in addition to informed consent of either parent or caretaker and of the child if aged 8 years or older: age 6 months to 18 years, term delivery (≥ 37 weeks); active/passive understanding of German. Steroid−/leukotriene receptor antagonist (LTRA)-naivety is defined as no use of inhaled or systemic corticosteroids or LTRA for at least four months prior to inclusion. Exclusion criteria for participants of the paediatric arm of ALLIANCE are: known inborn or perinatal pulmonary disease; pulmonary malformation; oxygen therapy after birth with a duration of more than 24 h; ventilator support or mechanical ventilation after birth; diagnosis of cystic fibrosis; primary ciliary dyskinesia; heart failure diagnosed after birth affecting pulmonary circulation; major respiratory diseases such as e.g. interstitial lung disease; and any current non-atopic comorbidities. Moreover, children are excluded from study visits and biomaterial collection in the case of fever of at least 38.5 °C during the last two weeks prior to the planned visit.

Childhood cases are specified as either having doctor-diagnosed wheeze during at least 2 occasions during the last 12 months (age < 6 years) or as having doctor-diagnosed asthma (age ≥ 6 years) with diagnosis according to current guidelines including lung function [10, 11]. Healthy controls are defined as children without wheeze or asthma and otherwise applying the same in- and exclusion criteria as mentioned above. Adolescent cases turning 18 years of age will automatically enter transition into the adult arm of ALLIANCE, with identical data and biomaterial collection as prior to transition.

### General in−/exclusion criteria for adults, definition of adult cases and controls

The following inclusion criteria apply in addition to informed consent for participants who are newly recruited during adulthood: age ≥ 18 years, active/passive understanding of German, and an established diagnosis of asthma according to current guidelines [10, 11]. Participants are allowed to be current or former smokers to avoid significant selection bias. Patients with asthma and a relevant smoking history are accurately screened for features distinguishing asthma from chronic obstructive pulmonary disease (COPD): age at onset, pattern and time course of symptoms, personal and family history, variable or persistent airflow limitation, lung function in symptom-free episodes, and severe hyperinflation [12]. If no clear distinction is possible and patients currently present with predominant features of COPD, i.e. relevant gas exchange impairment or hyperinflation, or signs/symptoms of chronic bronchitis or emphysema (if computer tomography scan available) [13], they are excluded from the study. Further exclusion criteria are: severe upper respiratory tract infection (URTI), or severe exacerbation during the last 4 weeks, ensuring that patients are in a stable phase of their disease [10, 11]. Controls had to be without any pulmonary disease but were allowed to have concurrent allergic rhinoconjunctivitis.

### Details on study design, recruitment, and follow-up

**Fig. 3:**
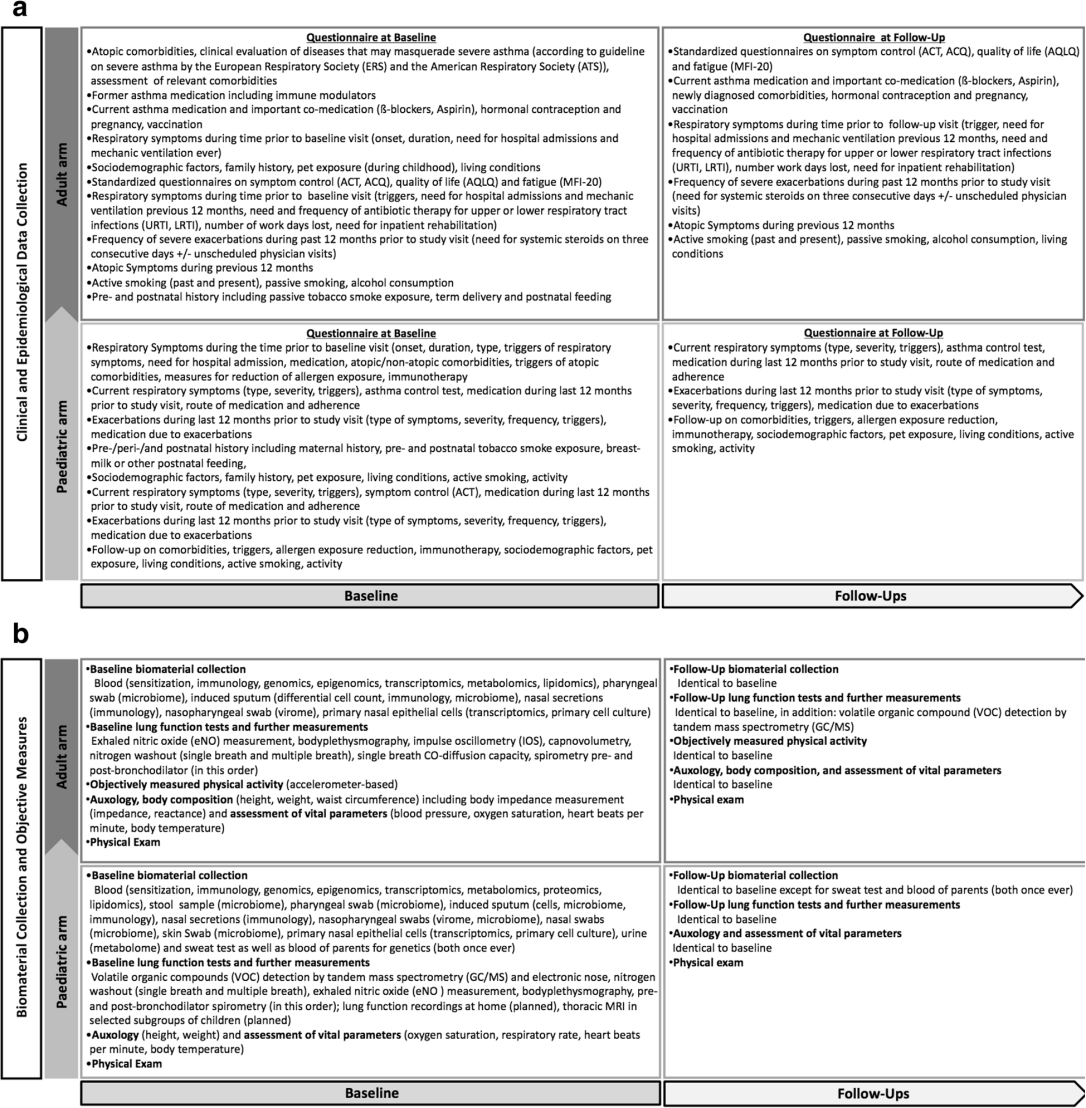
a,b: The DZL All Age Asthma Cohort (ALLIANCE): time-flow of recorded data (**a**), as well as of tests and procedures (**b**). See text for details.

The overall study design of the DZL Asthma Cohort is shown in Fig. 3 a and b, and detailed later in the text. Healthy controls are seen at the study centres once, yet their age distribution will allow comparisons across the whole age range of recruited cases. For cases, the design includes a first study visit at time of recruitment and regular yearly follow-up study visits afterwards.

Before recruitment, in- and exclusion criteria for eligible individuals are assessed by participating centres. In the case of children, this is additionally performed with screening leaflets used by participating primary care physicians with parents being asked for consent to transfer contact data to study centres prior to any communication between study centres and interested families. After information about the study and informed consent by study participants (> 8 years of age) and, if necessary, all care-takers, the study phase starts with recruitment and the first study visit of cases in each study centre. On this occasion, lung function measurements are performed, and questionnaires are used to assess important clinical and epidemiological data. Furthermore, extensive biomaterial collections are implemented (see Fig. 3 a and b for an overview over the time flow of recorded data as well as tests and procedures).

Recruitment of new cases for the paediatric arm of ALLIANCE is on-going. From September 2013 until and including December 2016, a total of *N* = 540 children (*n* = 415 cases, thereof *n* = 209 preschool wheezers and *n* = 206 childhood asthmatics, *n* = 125 healthy controls) were recruited as eligible study participants. For the adult arm, *N* = 255 study participants (*n* = 208 cases, *n* = 47 healthy controls) were included until and including December 2016 (see Fig. 2).

### How often have they been followed up?

After 12 months, all cases are invited for the first follow-up study visits at the study centres with identical measurements and data collection as during the baseline visit. On this occasion, a follow-up questionnaire is used to reassess important clinical and epidemiological items, likewise for all consecutive follow-up visits. In children, equivalent follow-up visits are performed every 12 months, while in adults, the schedule for deep-phenotyping follow-up visits is stretched.

Data collected during follow-up of cases has been added to the central database, quality controlled, and is reported here. In contrast to children, primary recruitment of adults was stopped in 2017 and only continued for those individuals who are already participants of the paediatric arm and turn adult during the time-course of the study.

Until the end of 2016, there was reasonable attrition during follow-up of cases (children and adults) as part of ALLIANCE (see Table 2). Among eligible children, i.e. individuals recruited until and including 2015 and with possible follow-up until and including 2016, we lost *n* = 52 of *n* = 324 children (16.0%) that should have completed study visits 12 months after initial recruitment, while this was the case for *n* = 17 of *n* = 172 (9.0%) 24 months, and for *n* = 4 of *n* = 20 (20.0%) 36 months after initial recruitment, respectively. Among eligible adults, *n* = 16 out of *n* = 169 (9.5%), who should have completed study visits 12 months and n = 3 out of *n* = 81 (3.7%), who should have completed study visits 24 months after initial recruitment, were lost over time. All individuals lost to follow-up were due to personal reasons. Further attrition during follow-up in 2017 and upcoming years will be evaluated regularly. Demographics of the *N* = 795 study participants of the DZL ALLIANCE cohort as part of the ALLIANCE database (at baseline for cases, only including eligible study participants) are displayed in Table 3 for both children and adults.

### What has been measured?

Data are derived from four sources: (a) hospital and medical records (used to extract information about wheeze and asthma diagnoses as well as in- and exclusion criteria), (b) questionnaires and structured interviews, (c) telephone interviews, and (d) objective measurements. A detailed description displaying the data collected in ALLIANCE for children and adults is presented in Table 1.

**Table 1:** Detailed description of collected data and measurements in the DZL All Age Asthma Cohort (ALLIANCE)

Data Collection (Hospital Records, Questionnaires, and Telephone Interviews)	Paediatric Arm	Adult Arm
Extraction of Routine data	Source: Questionnaires (Baseline and Follow-Up) • study participant: name, gender, city and country of birth, date of birth, birth mode, gestational age, birth weight, birth length, multiple birth, birth order, vaccinations, onset of puberty, physical activity, type of child care, desensitization, measures to reduce allergen exposure, asthma training • demographic and sociodemographic information: mother and father: names, country of birth, contact data (home address, telephone number, email address), country of birth (up to grand-parents), birth year, graduation, professional training, number of children and other persons in same household • feeding (breast-fed, hypoallergenic supplement) • family history (asthma, allergic rhinoconjunctivitis, atopic dermatitis, therapy, comorbidities assessed for parents and grand-parents as well as siblings) • families’ paediatrician name and address as well as of general practitioner	Source: Identification Questionnaire, Screening Questionnaire, Baseline Questionnaire (patient version), Baseline Questionnaire (physician version) • study participant: name, gender, city and country of birth, date of birth, birth mode, multiple birth, desensitization, measures to reduce allergen exposure, asthma training • demographic and sociodemographic information: contact data (home address, telephone number, email address), graduation, professional training, number of persons in same household (current and as child), country of birth of mother and father • feeding (breast-fed) • family history (asthma, assessed for parents and grand-parents as well as siblings) • patient’s pulmonologists name and address • vaccinations • atopy status according to study physician (allergic comorbidities, allergic sensitization without symptoms, no allergies), smoking status (current and former, cumulative pack years), alcohol consumption • comorbidities that might mask severe asthma; cardiovascular and neurologic comorbidities
Respiratory Symptoms	Source: Questionnaires (Baseline and Follow-Up) • Wheeze and cough ever prior to visit (age of onset, number, duration, severity, triggers, seasonal pattern of symptoms, treatment/medication) • Wheeze and cough during last 12 months prior to visit (number, duration, severity, triggers, seasonal pattern of symptoms, treatment/medication incl. Rout and technique as well as adherence, asthma control, assessed according to GINA [10] guidelines and by ACT [15, 37] • Wheeze and cough in relation to exacerbation (number, duration, severity, triggers of symptoms, treatment/medication) • Asthma Control assessed according to GINA [10], C-ACT, ACT [15, 37] • Allergic and non-allergic comorbidities (allergic rhinitis, atopic dermatitis, food allergy, age of onset, severity, triggers, seasonal pattern of symptoms, treatment/medication) • Medication (ever as well as resolution down to one month during last 12 months prior to routine study visit, resolution down to one day during last 4–5 weeks prior to exacerbation visits)	Source: Baseline and FollowUp Questionnaire (patient version), Baseline and FollowUp Questionnaire (physician version) ACT, ACQ, AQLQ, MFI-20 • first symptoms (age), first diagnosis and disease duration • Asthma Control assessed according to GINA [10], ACT [15], ACQ [16] • Health related quality of life assessed by AQLQ [17] • Fatigue assessed by MFI-20 [18] • Frequency of severe exacerbations, hospital admissions. Mechanical ventilation and asthma-related rehabilitation (ever and previous 12 months) • Allergic and non-allergic comorbidities (allergic rhinitis, atopic dermatitis, food allergy, age of onset, severity, triggers, seasonal pattern of symptoms, treatment/medication) • Current asthma medication (including current dosage of inhaled and oral corticosteroids), important co-medication (ß-blockers, Aspirin), former medication (including immune modulators), desensitization (former or current), work related symptoms
Environmental Exposures	Source: Questionnaires (Baseline and Follow-Up) • maternal warning signs sub partu (infection, fever, antibiotics or other medication, chorioamnionitis), signs of postpartal infection of child • maternal or paternal smoking during pregnancy and later, further active smoking in household • traffic exposure at home (major street) • mold exposure at home (assessment, refurbishment) • pet exposure at home (type of pet)	Source: Baseline Questionnaire (patient version), Follow-Up Questionnaire (patient version) • maternal or paternal smoking during pregnancy and later, further active smoking in household • mold exposure at home (assessment, refurbishment) • pet exposure in childhood (dogs)
Objective Measurements	Paediatric Arm	Adult Arm
Measurements of lung function and inflammation of airways, body composition and physical activity, imaging	1. Anthropometric data and vital parameters at study date: • Body weight and length, body temperature, heart beats per minute, respiratory rate, oxygen saturation 2. Exhaled breath measurement by electronic nose (e-nose) [21]: • Cyranose 320, Sensigent, Arrow Highway, USA • Main outcome parameters: patterns of volatile organic compounds (VOCs) 3. Exhaled breath measurement by gas chromatography-mass spectrometry (GC-MS) [22]: • Sampling device provided by Fraunhofer ITEM, Hannover • Main outcome parameters: patterns of volatile organic compounds (VOCs) by GC-MS 4. Single- and multiple breath washouts (nitrogen washout with 100% oxygen, O2) [19, 30]: • Ultrasonic flowmeter (EcoMedics AG, Duernten, Switzerland); single- and multiple-breath measurement, mouth-piece, filter, nasal clamp • Main outcome parameters (multiple breath): lung volume (functional residual capacity, FRC), ventilation inhomogeneity (lung clearance index, LCI) • Main outcome parameters (single breath): phase III and IV analysis (S_III_/ phase III slope, closing volume) 5. Measurement of exhaled nitric oxide (eNO) [20]: • Rapid-response chemoluminescence analyzer (CLD 88, EcoMedics AG, Duernten, Switzerland); single-breath manoeuvre, filter, no nasal clamp • Main outcome parameter: mean eNO, NO production (eNO x expiratory flow) 6. Spirometry and bodyplethysmography [28, 29]: • Jaeger MasterScreen Body, BD Carefusion, Germany; mouthpiece, filter, nasal clamp • Main outcome parameters: lung volumes (intrathoracic gas volume/functional residual capacity, FRC, total lung capacity, TLC, residual volume, RV), airway resistance, forced expiratory flows and volumes, each before and after bronchodilator 7. Planned: thoracic magnetic resonance imaging (tMRI)	1. Anthropometric data, vital parameters and body composition at study date: • Body weight and length, waist circumference, body temperature, heart beats per minute, respiratory rate, blood pressure, oxygen saturation, bioimpedance measurement (Nutri Plus, Data-Input GmbH, Darmstadt, Germany) • Main outcome parameters (bioimpedance): Resistance (50 kHz), Reactance (50 kHz), extra cellular mass/ body cell mass index (ECM/BCM Index) 2. Exhaled breath measurement by gas chromatography-mass spectrometry (GC-MS) [22]: • Sampling device provided by Fraunhofer ITEM, Hannover • Main outcome parameters: patterns of volatile organic compounds (VOCs) by gas chromatography-mass spectrometry (GC-MS) 3. Single and multiple breath washouts (nitrogen washout with 100% oxygen, O2) [19, 30]: • VMax ENCORE (Viasys Healthcare), Carefusion Germany; mouthpiece, filter, nasal clamp • Main outcome parameters (multiple breath): lung volume (functional residual capacity, FRC), ventilation inhomogeneity (lung clearance index, LCI) • Main outcome parameters (single breath): phase III and IV analysis (S_III_/ phase III slope, closing volume) 4. Measurement of exhaled nitric oxide (eNO) [20]: • Hand-held device (NIOX MINO, Circassia AB, Uppsala, Sweden); single breath manoeuvre, filter, no nasal clamp • Main outcome parameter: mean eNO 5. Bodyplethysmography [33]: • Jaeger MasterScreen Body, BD Carefusion, Germany; mouthpiece, filter, nasal clamp • Main outcome parameters: lung volumes (intrathoracic gas volume/functional residual capacity, FRC, total lung capacity, TLC, residual volume, RV), airway resistance, closing volume, closing capacity 6. Impulse Oscillometry (IOS) [23]: • Masterscreen IOS, BD Carefusion, Germany; mouthpiece, filter, nasal clamp • Main outcome parameters: Resistance (5 Hz, 20 Hz, Frequency dependent resistance FDR), Reactance (5 Hz, AX, Fres) 7. Capnovolumetry [24]: • Masterscreen Capno, Carefusion Germany; mouthpiece, filter, nasal clamp • Main outcome parameters (multiple breath): capnogram phase II and III analysis (Vm2550, SR23), dead space volumes • Main outcome parameters (single breath): capnogram phase II, III and IV analysis (Vm2550, SR23, S_III_/ phase III slope, closing volume), dead space volumes 8. Single Breath diffusing capacity (DLCO) [31]: • VMax ENCORE (Viasys Healthcare) and Jaeger MasterScreen Body, BD Carefusion Germany; mouthpiece, filter, nasal clamp • Main outcome parameters: transfer factor of the lung for carbon monoxide (TLCO) and related to alveolar volume (TLCO/VA) 9. Spirometry [29]: • VMax ENCORE (Viasys Healthcare) and Jaeger MasterScreen Body, BD Carefusion Germany; mouthpiece, filter, nasal clamp • Main outcome parameters: forced expiratory flows and volumes, each before and after bronchodilator
Objective Measurementscontinued	Paediatric Arm	Adult Arm
		10. Bronchial provocation (Methacholine) [38]: • VMax ENCORE (Viasys Healthcare), Jaeger MasterScreen Body and MasterScreen IOS, BD Carefusion Germany; mouthpiece, filter, nasal clamp • Main outcome parameter: PC_20_ FEV_1_, PC_40_ R5Hz, PC_40_ FRES 11. Daily Physical Activity (accelerometer based) [25, 26]: • SenseWear Armband, BodyMedia Inc. Pittsburgh (PA), USA • Main outcome parameters: steps per day (SPD) and indicators of daily energy expenditure
Sensitization	Specimen: peripheral blood 1. Measurement of total IgE, eosinophils in white blood cell count 2. Specific IgE (sIgE) measurement by chip technology (Immunocap ISAC 121) [39]: • Immunocap ISAC, Phadia, Uppsala, Sweden • Single components • Main outcome parameter: sensitization pattern to components 3. sIgE measurement by Sandwich-ELISA, Immunocap [40]: • Immunocap, Phadia, Uppsala, Sweden • Allergen extracts • Main outcome parameter: sensitization pattern to extracts 4. sIgE measurement by Immunoblot: • Euroimmun AG, Luebeck Germany • Allergen extracts • Main outcome parameter: sensitization pattern to extracts	Specimen: peripheral blood, skin 1. Measurement of total IgE, eosinophils in white blood cell count 2. Specific IgE (sIgE) measurement by chip technology (Immunocap ISAC 121) [39]: • Immunocap ISAC, Phadia, Uppsala, Sweden • Single components • Main outcome parameter: sensitization pattern to components 3. sIgE measurement by Immunoblot: • Euroimmun AG, Luebeck Germany • Allergen extracts • Main outcome parameter: sensitization pattern to extracts 4. Skin Prick Test • Allergopharma, Hamburg, Germany • Allergen Extracts • Main outcome parameter: sensitization pattern to extracts
Microbiome	Specimen: skin swabs, nasal swabs, (naso-)pharyngeal swabs, stool samples, induced sputum • Analysis by ultra-high-throughput sequencing • Analysis of microbiota in pharyngeal swab, stool and sputum samples	Specimen: pharyngeal swabs, induced sputum • Analysis by ultra-high-throughput sequencing • Analysis of microbiota in pharyngeal swab and sputum samples
Viruses, Virome	Specimen: nasopharyngeal swabs • Targeted analysis by virus-specific PCR • Virome by whole-genome sequencing	Specimen: nasopharyngeal swabs • Targeted analysis by virus-specific PCR • Virome by whole-genome sequencing
Immunology	Specimen: peripheral blood • White blood cell count, high-sensitive CRP, leukocyte subtypes by chip-cytometry [41] • Cytokines, cell-specific (blood cell sub-populations) analyses Specimen: nasal secretions • Cytokines Specimen: induced sputum • Cell subtypes • Cytokines	Specimen: peripheral blood • White blood cell count, high sensitive CRP, leukocyte subtypes by chip-cytometry [41] • Cytokines Specimen: nasal secretions • Cytokines Specimen: induced sputum • Cell subtypes • Cytokines
Genomics	Specimen: peripheral blood • DNA extraction from whole blood • Analysis of single nucleotide polymorphisms (SNPs)	Specimen: peripheral blood • DNA extraction from whole blood • Analysis of single nucleotide polymorphisms (SNPs)
Objective Measurementscontinued	Paediatric Arm	Adult Arm
Epigenomics	Specimen: peripheral blood (also parents of cases) • DNA extraction from whole blood, cell-specific (blood cell sub-populations) and tissue-specific analyses • Analysis of DNA methylation and histone modification	Specimen: peripheral blood • DNA extraction from whole blood • Analysis of DNA methylation and histone modification
Transcriptomics	Specimen: peripheral blood, primary nasal epithelial cells • RNA extraction from whole blood or primary cells, cell-specific (blood cell sub-populations) and tissue-specific analyses • Analysis of RNA by array analysis and of candidates by real-time PCR (RT-PCR)	Specimen: peripheral blood, primary nasal epithelial cells • RNA extraction from whole blood or primary cells • Analysis of RNA by array analysis and of candidates by real-time PCR (RT-PCR)
Metabolomics	Specimen: peripheral blood, urine • Targeted and non-targeted metabolomics from serum and urine samples • Analysis of several hundred human metabolites by HPLC-MS or MS/MS	Specimen: peripheral blood • Targeted and non-targeted metabolomics from serum samples • Analysis of several hundred human metabolites by HPLC-MS or MS/MS
Lipidomics	Specimen: peripheral blood • Targeted analysis of lipids in peripheral blood • Analysis by LC-MS/MS	Specimen: peripheral blood • Targeted analysis of lipids in peripheral blood • Analysis by LC-MS/MS
Proteomics	Specimen: peripheral blood • Targeted analysis of proteome in peripheral blood

**Table 2:** Completion Rates of Annual Follow-Ups of Cases of the DZL All Age Asthma Cohort (ALLIANCE)

Date: 31/12/2016	Completed Study Visits	Documented Drop-Outs	Sum	Attrition
Pediatric Arm				
- Baseline	415	NA	NA	NA
- 12 months	272	52	324	16.1%
- 24 months	172	17	189	9.0%
- 36 months	16	4	20	20.0%
Adult Arm				
- Baseline	208	NA	NA	NA
- 12 months	153	16	169	9.5%
- 24 months	78	3	81	3.7%

**Table 3:** Baseline demographics of study participants (cases and controls) in the DZL All Age Asthma Cohort (ALLIANCE)

Demographic data – Paediatric Arm, N = 540	Median	Interquartile range
Age at baseline study date, yrs	6.6	3.1–11.4
	Number	Proportion (%)
Sex, male	341	63.1
Current smoker^a^	2	0.9
Demographic data – Adult Arm, N = 255	Median	Interquartile Range
Age at baseline study date, yrs	52.0	43.0–64.0
	Number	Proportion (%)
Sex, male	117	45.9
Smoker^b^	60	23.5
Non-smoker^b^	195	76.5

^a^In the pediatric arm only children aged 8 years or more were asked for their smoking habits (*n* = 224). Data were missing for n = 12 children

^b^In the adult arm current smokers and former smokers with at least 10 pack years (PY) smoking history were defined as ‘smokers’ (n = 16 (26.7%) and *n* = 44 (73.3%), respectively); non-smokers could be either never-smokers, or former smokers with less than 10PY. Among all adult former smokers and current smokers, mean (range) cumulative tobacco exposure was 9.5 PY (4–20)

### Questionnaires, structured interviews, telephone interviews

Questionnaires are used to collect information on health conditions, with an emphasis on respiratory and atopic symptoms, infections, and sociodemographic and environmental exposures. In children, a baseline questionnaire further collects information on pre- and perinatal risk factors including family past medical history. During follow-up, a further questionnaire assesses the study participants’ history during the period prior to the study visit. Both questionnaires used in the paediatric arm contain previously validated questions from the International Study of Asthma and Allergies in Childhood (ISAAC) [14]. Questionnaires in the adult arm cover the same topics and adopted the wording of the questions in an appropriate way. However, while some sections, e.g. pre- and postnatal risk factors, had to be shortened due to recall bias, other questions e.g. on comorbidities and co-medication were included and further validated questionnaires are distributed separately (Asthma Control Test, ACT [15]; Asthma Control Questionnaire, ACQ [16]; Asthma Quality of Life Questionnaire, AQLQ [17]; Multidimensional Fatigue Inventory, MFI-20 [18]). All questionnaires are used with little variation since 2012. For children, all questionnaires are programmed as an online database for data entry during interview. For adults, print-out versions of the structured questionnaires are primarily filled in by the patient and re-checked by the responsible study physician. Additional telephone interviews are used within a time window of up to one week after scheduled meetings to collect epidemiological data with use of baseline or follow-up questionnaires whenever these could not be completed during study visits.

### Objective measurements

For ALLIANCE participants, objective measurements include routine lung function tests such as spirometry and bodyplethysmography, but also more specialized techniques such as single and multiple breath washout measurements (SBW and MBW, respectively) [19] to assess small airway function and ventilation inhomogeneity. This is complemented by qualitative and quantitative assessments of airway inflammation using exhaled nitric oxide (eNO) measurements [20] as well as analyses of exhaled breath with an electronic nose (e-nose, in children only) [21] and with gas chromatography – mass spectrometry (GC/MS) [22]. In adults, assessment of small airway function is additionally performed by impulse oscillometry (IOS) [23] and capnovolumetry [24]. Objective lung function measurements in adults are further complemented by whole body bioimpedance measurement and objectively measured daily physical activity [25–27]. All measurements are performed according to current guidelines by the European Respiratory Society (ERS) and the American Thoracic Society (ATS) [19, 20, 28–33].

For ALLIANCE study participants and healthy controls, biomaterial collection during study visits include: peripheral blood samples (inflammatory markers, cell subpopulations and cytokines, sensitization pattern, metabolomics, proteomics (in children only), lipidomics, genomics, transcriptomics, and epigenomics); skin, nasal, pharyngeal, and nasopharyngeal swabs (microbiome, virome); collection of nasal secretions (cytokines); primary epithelial cell scrapings (transcriptome, primary epithelial cell culture); induced sputum samples (cytokines, microbiome, cell subsets); and urine (metabolomics); as well as stool samples (microbiome) only in children. Parents of participating children are also asked for a blood sample (genomics).

## Discussion

The ALL Age Asthma Cohort is a large integrative and interdisciplinary framework containing data on *n* = 623 patients with wheeze and asthma and *n* = 172 healthy controls at baseline. With its comprehensive, standardized molecular approach in a prospective and identical fashion for children and adults, ALLIANCE aims at (a) identification of biomarkers and predictors to decode the complex mechanisms that may underlie the development of distinct childhood wheeze and asthma trajectories as well as their transition into and further course during adulthood, and at (b) translation of this into clinical practice for individual patients.

In addition to the comprehensive and prospective approach across all ages, ALLIANCE has several methodological strengths, which are its prime features: the prospective, observational approach with standardized data and comprehensive biomaterial collection across all ages during each study visit in a multi-centre setting to delineate the different wheeze and asthma phenotypes; the accurate and standardized measurements of lung function and markers of airway inflammation, and the stringent quality control measures involved for every single data point.

In a broad approach, ALLIANCE aims at decoding the complex mechanisms that may underlie the development of distinct childhood wheeze and asthma phenotypes and their transition into adulthood as well as later adult asthma phenotypes. As we hypothesize that specific molecular phenotypes are associated with distinct wheeze/asthma trajectories, this includes the meticulous longitudinal collection of routine clinical and epidemiological data and exhaustive examination of phenotype components that go beyond medical chart data. Thereby, underlying mechanisms as well as predictors and biomarkers for such traits can be identified. For children, this is being applied to study both participants with established diagnoses and those possibly on controller therapy, as well as in children which are steroid- and LTRA-naïve, ideally at the earliest possible disease state.

Thus, we follow a thorough and comprehensive deep phenotyping approach in cases and healthy controls. This includes extensive quality control during data collection. In this respect, besides the fact that all measurements are performed according to current guidelines where available, more than 50 standard operating procedures (SOPs) for data or biomaterial collection as well as their processing, shipment, and analyses have been developed. All data collection is being performed in a standardized way at any age, using the same technique and equipment on every subsequent occasion in the same order and according to the numerous harmonized SOPs. Adherence to these SOPs is ensured by regular field and lab audits. In addition, structured site visits and team meetings across all involved centres are performed regularly.

To explain the association of lung mechanics during early and late childhood as well as adult age with wheeze and asthma phenotypes as part of a physiological phenotyping, overall lung function, small airway function, and markers for ventilation inhomogeneity are measured. This is complemented by qualitative and quantitative assessments of airway inflammation and analyses of exhaled breath. Lung function tests are performed at several time points covering important phases of lung development (further growth of airways and alveoli) during early and later childhood [34]. Relevant confounders such as physical activity and auxological as well as developmental features are prospectively assessed and will be included in all analyses. All lung function and eNO measurements are performed according to the latest ERS/ATS recommendations, if available [19, 20, 28, 29, 31–33].

There is still limited knowledge on the genetic impact on childhood wheeze and asthma as well as adult asthma. This is particularly true for cell- or target-tissue specific up- or downstream events involving epigenetic control as well as gene expression. Therefore, in addition to genotyping, the assessment of transcriptomics and epigenomics both in whole blood, cell-specific (blood cell sub-populations) and tissue-specific analyses (primary cells of the upper airways and biosamples from induced sputum) is part of the deep phenotyping strategy of ALLIANCE. Another hallmark hypothesis of the consortium is that individuals at risk for exacerbations can be identified by clinical and molecular biomarkers, which might become novel targets for therapy and secondary prevention. Therefore, exacerbations will be assessed by numbers (children and adults) as well as triggers, severity and clinical features (children only) via questionnaires at all visits.

So far, our attrition rates are comparable to other studies [35, 36]. This may be due to the regular follow-up in the clinical setting. The drawback of such a detailed, time-consuming longitudinal study is that it can only be done on a limited number of participants due to the significant workload regarding logistics, data collection and analyses. This extensive biomaterial collection and the number of different analyses and hypotheses is also the reason, why a single power calculation for the whole cohort is not feasible. Sample size for both study arms is therefore based on pragmatism and clinical experience from comparable cohorts.

A significant limitation of ALLIANCE is the inclusion of cases into the study earliest at the time-point of disease manifestation, which renders assessment of prior or even causal determinants acting before disease onset impossible. Such assessments can only be performed by population-based birth cohort studies. Moreover, in contrast to cross-sectional comparisons, the lack of prospective follow-up of healthy controls identical to the setup performed for cases hinders comparability over time across both strata. Lastly, data on environmental exposures (e.g. allergen or microbial exposure) are only collected by questionnaires and not by objective sampling and measurement. Methodology in both study arms was harmonized as far as possible. However, both study arms are also used to answer individual research questions of paediatric and adult pulmonologists, while other measurements are not feasible or scientifically reasonable. Therefore some objective measurements are performed in one arm only.

So far, all involved study participants for the paediatric arm were recruited in participating hospital centres and private practices of registered paediatricians in the regions of Luebeck, Hannover, Munich, and for the adult arm in participating hospitals and research centres in Grosshansdorf and Borstel near Hamburg. Transition clinics will be established in all three sites to secure continuous follow-up across the otherwise often neglected gap between paediatric and adult pneumologist care. New study centres for the paediatric arm (Marburg, Cologne) have started recruitment in 2017. While the main reasons for non-participation especially of early-onset wheezers is lacking information about the study as well as the amount of data and biomaterial that is being collected, all participants are likely to be biased towards a well-educated middle-class population, which is the case with numerous studies bringing along detailed data collection. Moreover, this is an observational study within a clinical setting with desirable results to be applied in such environments. Hence, results will be less applicable for the general population than for individuals presenting to seek medical care in the case of wheeze and asthma.

In this clinical context, ALLIANCE offers improved endo-phenotyping in children and adults identically to delineate different underlying pathophysiological pathways across asthma phenotypes in a longitudinal way. With its standardised and unique dataset of detailed epidemiological, physiological, and deep phenotyping of a comprehensive range of biomaterials across all ages in a considerable number of study participants, it will enable to decode mechanisms underlying the asthma syndrome, as well as their translation to well-defined patient groups and possibly even the individual patient.

## Abbreviations

(HP)LC: (high performance) liquid chromatography
ACQ: Asthma Control Questionnaire
ACT: Asthma Control Test
ALLIANCE: All Age Asthma Cohort
AQLQ: Asthma Quality of Life Questionnaire
ARCN: Airway Research Centre North
ATS: American Thoracic Society
BMBF: Bundesministerium für Bildung und Forschung (German Federal Ministry of Education and Research)
BREATH: Biomedical Research in Endstage and Obstructive Lung Disease Hannover
COPD: Chronic obstructive pulmonary disease
CPC-M: Comprehensive Pneumology Centre Munich
DA AA: Disease Area Asthma Allergy
DZL: Deutsches Zentrum für Lungenforschung (German Centre for Lung Research)
ELISA: Enzyme-linked immunosorbent assay
eNO: Exhaled nitric oxide
ERS: European Respiratory Society
GC/MS: Tandem mass spectrometry
GINA: Global Initiative for Asthma
IOS: Impulse oscillometry
ISAAC: International study of Asthma and Allergy in Childhood
LRTI: Lower respiratory tract infection
LTRA: Leukotriene-receptor antagonist
MFI-20: Multidimensional Fatigue Inventory
sIgE: Specific immunoglobulin E
SOP: Standard operating procedure
UGMLC: Universities of Giessen and Marburg Lung Centre
URTI: Upper respiratory tract infection
