# Correction to: The all age asthma cohort (ALLIANCE) - from early beginnings to chronic disease: a longitudinal cohort study

**DOI:** 10.1186/s12890-018-0717-2

**Published:** 2018-11-07

**Authors:** Oliver Fuchs, Thomas Bahmer, Markus Weckmann, Anna-Maria Dittrich, Bianca Schaub, Barbara Rösler, Christine Happle, Folke Brinkmann, Isabell Ricklefs, Inke R. König, Henrik Watz, Klaus F. Rabe, Matthias V. Kopp, Gesine Hansen, Erika von Mutius, the ALLIANCE Study Group as part of the German Centre for Lung Research (DZL)

**Affiliations:** 1 Dr von Hauner Children’s Hospital, Ludwig Maximilians University, Munich, Germany; 2 Department of Paediatric Respiratory Medicine, Inselspital, University Children’s Hospital of Bern, University of Bern, Bern, Switzerland; 3 Division of Pediatric Pulmonology and Allergology, University Children’s Hospital, Luebeck, Germany; 4 LungenClinic Grosshansdorf GmbH, Grosshansdorf, Germany; 5 Department of Paediatric Pneumology, Allergology and Neonatology, Hannover Medical School, Hannover, Germany; 6 Department of Paediatric Pneumology, University Children’s Hospital, Ruhr-University Bochum, Bochum, Germany; 7 Institute for Medical Biometry and Statistics, University Luebeck, University Medical Centre Schleswig-Holstein, Campus Luebeck, Luebeck, Germany; 8 Pulmonary Research Institute at LungenClinic Grosshansdorf, Grosshansdorf, Germany; 9 Institut für Asthma- und Allergieprävention (IAP), Helmholtz Zentrum Munich, Deutsches Forschungszentrum für Gesundheit und Umwelt (GmbH), Munich, Germany; 10 Comprehensive Pneumology Center - Munich (CPC-M), German Center for Lung Research (DZL), Munich, Germany; 11 Airway Research Center North (ARCN), German Center for Lung Research (DZL), Grosshansdorf, Germany; 12 Biomedical Research in Endstage and Obstructive Lung Disease Hannover, Hannover, Germany

## Correction

Following publication of the original article [1], the author flagged aspects of the article that affected readability of some of the article’s scientific content.

Some aspects pertained to formatting, while some pertained to content.

The formatting and content of the article [1] has been amended to resolve the flagged aspects.

Regarding content, please note that this update has been made to the content of the article [1]:

The title of the leftmost column of Table 1 on page 6 has been amended to: **Objective Measurements**.
